# Supplementary material for: Novel Driver Strength Index highlights important cancer genes in TCGA PanCanAtlas patients
Source: PeerJ. 2022 Aug 11;10:e13860. doi: 10.7717/peerj.13860 (PMC9375969; doi:10.7717/peerj.13860)
Supplement: Supplemental Information 2 [file peerj-10-13860-s002.zip › COHORTS/genes plots/cohorts/2021_8_16_14_9_distribution_cohorts_PCPG.pdf]

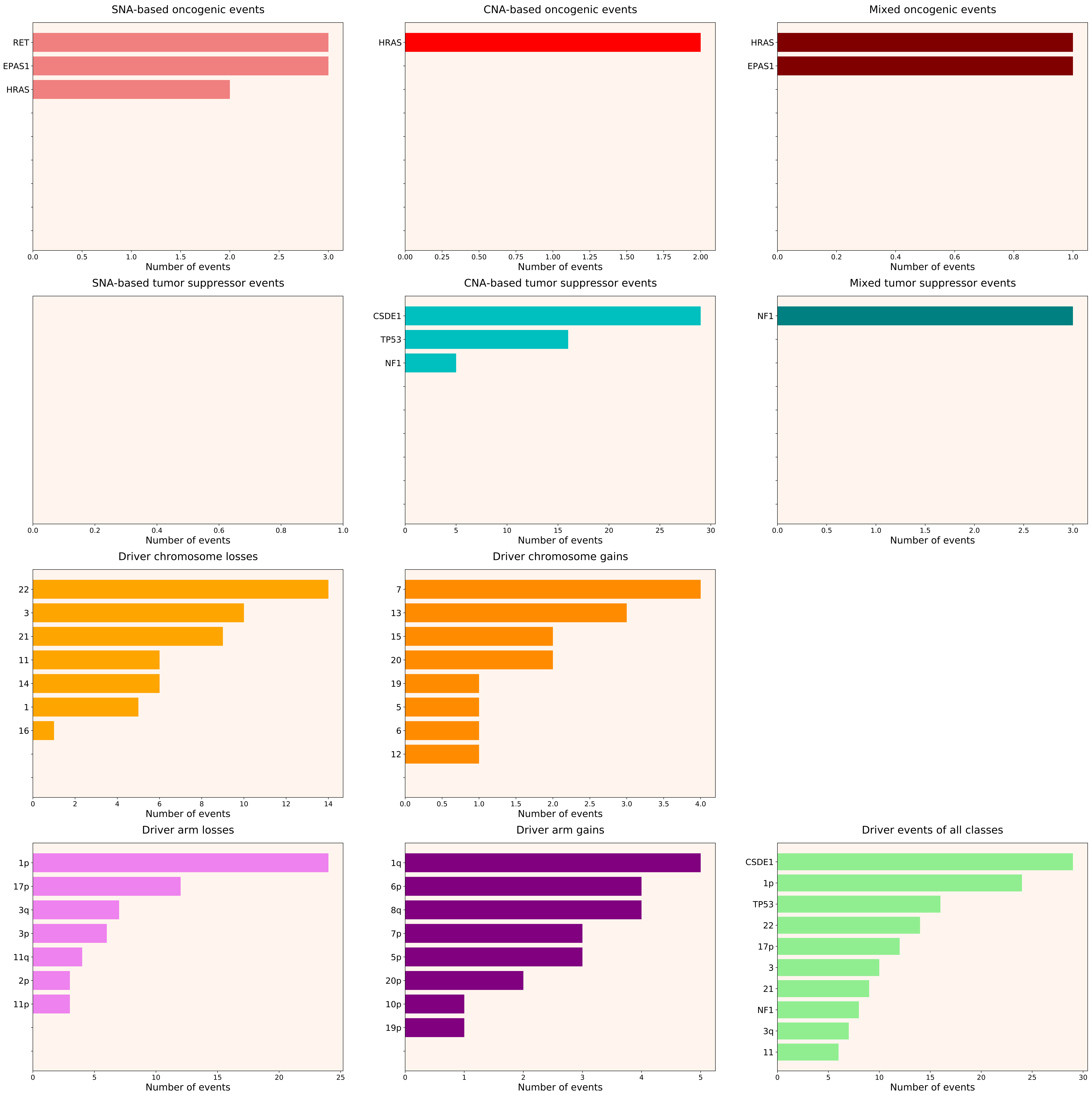
SNA-based oncogenic events
RET
EPAS1
HRAS
Number of events
CNA-based oncogenic events
HRAS
Number of events
Mixed oncogenic events
HRAS
EPAS1
Number of events
SNA-based tumor suppressor events
Number of events
CNA-based tumor suppressor events
CSDE1
TP53
NF1
Number of events
Mixed tumor suppressor events
NF1
Number of events
Driver chromosome losses
22
3
21
11
14
1
16
Number of events
Driver chromosome gains
7
13
15
20
19
5
6
12
Number of events
Driver arm losses
1p
17p
3q
3p
11q
2p
11p
Number of events
Driver arm gains
1q
6p
8q
7p
5p
20p
10p
19p
Number of events
Driver events of all classes
CSDE1
1p
TP53
22
17p
3
21
NF1
3q
11
Number of events
